# Prevalence and description of digital device use among preschool children: A cross-sectional study in Kota Setar District, Kedah

**DOI:** 10.51866/oa.25

**Published:** 2022-10-27

**Authors:** Tanusha Nathan, Leelavathi Muthupalaniappen, Noor Azimah Muhammad

**Affiliations:** 1 MMed Fam Med, Department of Family Medicine, Faculty of Medicine14th Floor, Pre-clinical Block, Universiti, Kebangsaan Malaysia Medical Center, Jalan Yaacob Latif, Bandar, Tun Razak, Cheras, Kuala Lumpur, Malaysia. Email: drleelaraj@gmail.com; 2 MMed Fam Med, Family Medicine Specialist, Kelinik Kesihatan Kampung Lalang, Kampung Lalang, Baling, Kedah, Malaysia.; 3 MMed Fam Med, Department of Family Medicine, Faculty of Medicine, 14th Floor, Pre-clinical Block, Universiti, Kebangsaan Malaysia Medical Center, Jalan Yaacob Latif, Bandar, Tun Razak, Cheras, Kuala Lumpur, Malaysia.

**Keywords:** Child, Preschool, Prevalence, Smartphone, Screen time

## Abstract

**Introduction:**

Digital device helps children enhance academic, cognitive and psychomotor skills. However, prolonged use causes physical inactivity, poor interpersonal skills and communication problems. Information on digital device use among young children in Malaysia is currently limited. Hence, this study aimed to determine the prevalence of digital device utilisation among preschool children in Kota Setar District, Kedah.

**Method:**

A cross-sectional study at government preschools in Kota Setar District was conducted from February to April 2020. Selection of preschools and students was done using multistage simple randomisation. A self-administered questionnaire containing demographic and digital device use details was filled by parents.

**Results:**

The prevalence of digital device use among preschool children was 95.9% and mostly used smartphones (94.2%). Most children (95%) did not own the device, and usage was under supervision (95.7%). The reason for supervision was to prevent exposure to inappropriate content (70.5%). The common reasons for allowing digital device use were for educational (37.4%) and entertainment purposes (36%) through videos (30.9%) and games (30.2%). Approximately 21.5% and 50.3% of the children spent more than 1 and 2 hours on digital devices during weekdays and weekends, respectively.

**Conclusion:**

The prevalence of digital device use among the preschool children in Kota Setar District was very high. Most of them used digital devices for educational and entertainment purposes under parental supervision. However, some exceeded the recommended screen time on weekends. These findings could promote awareness of digital device use among young children and help design public health awareness programmes and future policies.

## Introduction

Digital devices are physical units of digital equipment, which include smartphones, smart watches, tablets, desktops, laptops and computers.^1^ These devices have become an essential necessity in daily living. Their use has permeated the daily activities of children, gaining importance in education, entertainment and communication, and has become every child’s dream toy.^2,3^ Approximately half of children (52%) have access to digital devices, such as smartphones (41%), video iPods (21%) or tablets (8%), with majority of parents (98%) allowing their children to use these devices.^4-6^

Digital device use has both positive and negative impacts in children.^7,8^ Academically, digital device use has a positive impact on preschool children towards learning by increasing alphabet recognition and enhancing reading, early language and mathematical skills. Cognitively, it enhances visual intelligence skills and helps children develop psychomotor skills. Meanwhile, the negative impacts include physical inactivity leading to increased risks of musculoskeletal problems and obesity. Psychologically, there is a risk of developing addictive disorders, problems in differentiating fantasy from reality, depression, aggressive and violent behaviours, conduct problems, hyperactivity and inattention.^9^ High digital device usage is also associated with social isolation, poor interpersonal skills, communication problems and decreased quality family time.^10^ Apart from using digital devices for education, preschool children also use them for playing games and watching videos.^3,11^

Globally, approximately one-third of preschool children have access to digital devices, and majority of them exceed the recommended screen time.^3,12,13^ Excessive digital device utilisation among young children causes varying problems, including obesity, poor sleep, behavioural problems and lack of attention. Currently, there are limited data on digital device use among preschool children in Malaysia; most studies on digital device use among young children were conducted in developed countries. Hence, this study aimed to evaluate the prevalence and description of digital device use among preschool children locally to address this gap. This information could be used to plan awareness programmes and future policies.

## Methods

A cross-sectional study was conducted from February to April 2020 among parents of preschool children aged 4–6 years in Kota Setar District, Kedah. Parents were defined as biological parents (either mothers or fathers) or main caregivers (e.g. step-parents, grandparents, uncles or aunts). The sample size was calculated using the Epi calculator based on an expected frequency of 50%, acceptable margin of error 5% and added possibility rate of incomplete or unreturned questionnaires of 20%. The sample size required was 145 individuals. The use of an expected frequency of 50% was based on an educated guess, as no previous local study has evaluated the prevalence of digital device use among preschool children.

A self-administered questionnaire was adopted and used with permission from two earlier studies.^3,6^ This questionnaire contained information on parents’ demographic profiles (10 items) and description of their children’s digital device use (9 items). The main measures in the questionnaire were digital device use, common devices used, device use duration, reason for use, and use supervision. Adaptation and content validation were performed by two family medicine specialists (one with a PhD in community child and adolescent health) and a trainee in family medicine. Content validation was conducted by examining each item in the questionnaire to assess whether it fairly represented the measure it was intended for and whether it was suitable for local use. All items were accepted as suitable, and the final questionnaire was then translated (forwards and backwards) into the local language (Bahasa Malaysia) by two linguists and subjected to face validation. For face validation, the questionnaire was distributed to 20 parents from a different kindergarten over 1 week. They were given time to fill in the questionnaire in the presence of the researcher and instructed to provide feedback if there was any difficulty in answering the questionnaire. All 20 parents provided feedback that they easily understood the questionnaire and were able to answer all sections without difficulty; hence, no changes were made to the questionnaire. Smartphones, touch-screen tablets (e.g. iPad), laptops and desktop computers were considered digital devices. Televisions were excluded from the list to avoid duplication of information, as many households could view programmes screened on the television using digital devices.

The participants were selected using multistage simple randomisation. Initially, 15 preschools were randomly selected from 75 preschools registered under the Ministry of Education in Kota Setar District using the ballot method. From each preschool, 15 students were randomly selected using computer-generated numbers based on the student registry. These children then received an envelope for their parents containing the information sheet, consent form and questionnaire through their class teachers. They were requested to return the answered questionnaire within a week. The class teachers reminded the students who did not return the questionnaire. Thereafter, the questionnaires were collected from the teachers. The parents who answered that their child did not use any digital devices were instructed to skip the section on the description of digital device use and return the questionnaire. Those who were unable to understand the national language, Bahasa Melayu, or did not consent were excluded. Data were entered into SPSS version 26. (IBM Released 2019. IBM SPSS Statistics for Windows, Version 26.0. Armonk, NY: IBM Corp) and descriptive analysis was used to evaluate the prevalence and description of digital device use among the preschool children. This study was approved by the research ethics committee of Universiti Kebangsaan Malaysia (FF-2019-381) and Kota Setar District Education Office.

## Results

All 145 parents completed the questionnaires, which were returned through their preschool children. The prevalence of digital device use among the children was 95.9% (n=139), with a mean age at exposure of 3.9 years (SD=1.25). Most respondents were mothers (66.2%, n=92), had tertiary education (54.7%, n=76), had three or more children (64.6%, n=90) and belong to the middle- and higher-income groups (60.4%, n=90). All demographic features of the participants are shown in **Table 1**.

**Table 1:** Demographic characteristics of parents whose preschool children use digital devices (n=139).

Participant characteristics	n (%)
*Ethnicity*	
Malay	116 (83.5%)
Chinese	13 (9.4%)
Indian	9 (6.5%)
Others	1 (0.7%)
*Relationship with the child*	
Mother	92 (66.2%)
Father	43 (30.9%)
Others (legal guardians/grandparents)	4 (2.9%)
*Total number of children*	
≥3	90 (64.6%)
<3	49 (35.3%)
**Educational level**	
*School*	
With formal schooling	62 (44.6%)
Without formal schooling	1 (0.7%)
*Tertiary*	
Diploma	43 (30.9%)
Degree	24 (17.3%)
Postgraduate degree	9 (6.5%)
**Educational level of the spouse**	
*School*	
With formal schooling	84 (60.4%)
Without formal schooling	1 (0.7%)
*Tertiary*	
Diploma	33 (23.7%)
Degree	16 (11.5%)
Postgraduate degree	5 (3.6%)
*Marital status*	
Married	136 (97.8%)
Divorced	3 (2.2%)
*Occupation*	
Private/government employee	85 (61.2%)
Unemployed (homemaker)	34 (24.5%)
Self-employed	20 (14.4%)
*Occupation of the spouse*	
Private/government employee	86 (61.9%)
Self-employed	47 (33.8%)
Unemployed/homemaker	6 (4.3%)
Total income	
Less than RM 2500 (Low)	55 (39.6%)
RM 2500 to 10000 (Middle)	78 (56.1%)
More than RM 15000 (High)	6 (4.3%)

The most common type of digital device used by the children was smartphones (94.2%, n=131), followed by tablets (32.4%, n=45). Majority (95%, n=132) of them did not have their own device, and device use was under parental supervision (95.7%, n=133). Majority of the parents supervised the usage to ensure that their child was not exposed to inappropriate content (70.5%, n=98) and could benefit from it (54%, n=75) (**Table 2**).

**Table 2:** Description of digital device use among preschool children.

Device description and usage	n (%)
*Own device*	
No	132 (95.0%)
Yes	7 (5.0%)
*Type of device used*	
Smartphone	131 (94.2%)
Tablet	45 (32.4%)
Laptop	24(17.3%)
Desktop computer	17 (12.2%)
*Supervision*	
Under supervision	133 (95-7%)
On their own	5 (3.6%)
Together with friends	5 (3.6%)
*Reason for supervision*	
To make sure he/she is not exposed to inappropriate content	98 (70.5%)
It helps him/her to benefit more from it	75 (54.0%)
He/she needs help to use it	48 (34.5%)
He/she asks me to use it together with him/her	38 (27.3%)
I happen to be in the same room with my child	37 (26.6%)
It is our time together	28 (20.1%)
I enjoy using it together	11 (7.9%)
Other reasons	4 (2.9%)
I do not use devices together with my child	3 (2.2%)

The digital device use duration during weekdays was mostly between 30 minutes and 1 hour. During weekdays, approximately 21.5% (n=30) of the children spent 1-2 hours on digital devices; this figure increased to 50.3% (n=70) on weekends. Approximately 29.4% (n=41, on weekdays) to 56.8% (n=79, on weekends) spent >1 hour on digital devices. Digital devices were often used at home and outdoors (**Table 3**).

**Table 3:** Duration and place of digital device use during weekdays and weekends.

Duration (n=139)	None n (%)	30 minutes to 1 hour n (%)	1 to 2 hours n (%)	More than 2 hours n (%)
**Place and day of the week**				
*Weekdays*				
Preschool	122 (87.8%)	14 (10.1%)	2 (1.4%)	1 (0.7%)
Babysitter	108 (77.7%)	24 (17.3%)	4 (2.9%)	3 (2.2%)
Home	67 (48.2%)	56 (40.3%)	10 (7.2%)	6 (4.3%)
Outdoors	74 (53.2%)	50 (36.0%)	14 (10.1%)	1 (0.7%)
*Weekends*				
Preschool	139 (100%)	0	0	0
Babysitter	117 (84.2%)	11 (7.9%)	7 (5.0%)	4 (2.9%)
Home	66 (47.5%)	35 (25.2%)	35 (25.2%)	3 (2.2%)
Outdoors	78 (56.1%)	31 (22.3%)	28 (20.1%)	2 (1.4%)

The common activities on digital devices were video watching (30.9%, n=43), game playing (30.2%, n=42) and educational learning (22.3%, n=31). Approximately 20% (n=28) used digital devices for reading (**Table 4**).

**Table 4:** Common activities of preschool children on digital devices.

Common activities on digital devices	Always n(%)	Occasionally n(%)	Rarely n(%)	Never n(%)
Watch movies/videos/shows	43 (30.9%)	52 (37.4%)	25 (18.0%)	19 (13.7%)
Play games for fun	42 (30.2%)	52 (37.4%)	32 (23.0%)	32 (23.0%)
Play games to learn	31 (22.3%)	64 (46.0%)	33 (23.7%)	11 (7.9%)
Read/look at books	28 (20.1%)	56 (40.3%)	26 (18.7%)	29 (20.9%)
Phone/video calls	21 (14.4%)	29 (20.9%)	51 (36.7%)	39 (28.1%)
Listen to music	19 (13.7%)	43 (30.9%)	36 (25.9%)	41 (29.5%)
Take photographs/videos	13 (9.4%)	49 (35.3%)	46 (33.1%)	31 (22.3%)

The common reasons for allowing digital device use were educational (37.4%, n=52) and entertainment purposes (36.0%, n=50) and boredom avoidance (29.5%, n=4l). Approximately one-quarter of the parents allowed digital device use to keep their child safe and out of trouble (27%, n=38) (**Table 5**).

**Table 5:** Reasons for allowing digital device use among preschool children.

Use of digital device for the following situations	Always n (%)	Occasionally n (%)	Rarely n (%)	Never n (%)
To learn something	52 (37.4%)	67 (48.2%)	11 (7.9%)	9 (6.5%)
To be entertained	50 (36.0%)	53 (38.1%)	30 (21.6%)	6 (4.3%)
Because he/she is bored	41 (29.5%)	57 (41.0%)	31 (22.3%)	10 (7.2%)
So he/she can be active (e.g. dance)	26 (18.7%)	38 (27.3%)	52 (37.4%)	23 (16.5%)
So he/she stays safe and out of trouble	38 (27.3%)	67 (48.2%)	25 (18.0%)	9 (6.5%)
To connect with other family members	32 (23.0%)	54 (38.8%)	34 (24.5%)	19 (13.7%)
So I can get things done	20 (14.4%)	63 (45.3%)	37 (26.6%)	19 (13.7%)
To calm him/her down	18 (12.9%)	51 (36.7%)	46 (33.1%)	24 (17.3%)
To go to sleep	4 (2.9%)	16 (11.5%)	23 (16.5%)	96 (69.1%)

## Discussion

The prevalence of digital device use among preschool children in advanced countries such as Russia and the United States of America (USA) is approximately 92%.^2,6^ Our study found a similar prevalence of 95.9%, which indicates that majority of preschool children have access to and are using digital devices. In Singapore, a lower prevalence (25%) of smartphone and touch-screen tablet usage was observed among children;^7^ however, this study was conducted in the year 20l5 and the current figures may be higher. Herein, the average age at the initial exposure of the preschool children to digital devices was 3.9 years. Children in the USA and Korea mostly started using digital devices between the age of six months and five years.^3,l4^ Although the average age at initiation of digital device usage locally was more than six months, it remains within the same age range. This is probably because in the Malaysian school system, preschool starts at the age of three years and primary school at the age of seven years. Hence, the preschool population in previous studies was younger, as their preschool cut-off age was lower. This shows that digital devices are incorporated into the lives of children at a very young age. The fact that digital devices are becoming more affordable, better internet availability and the sociocultural background of young parents may contribute to the early age of initiation.^l5,l6^ The American and Canadian Academy of Paediatrics recommends the avoidance of screen-based devices (except for video chats) for children aged <18 months. For those aged 18–24 months, high-quality programmes may be watched with parents, while for those aged 2–5 years, quality programmes are advised to be watched for only 1 hour daily. However, the guideline does not specify the device type but focuses on the screen time.^17,18^

Most parents had school education (both primary and secondary), while one-third had tertiary education. More than half of the families (56.1%) were within the middle-income bracket. Accordingly, digital devices and internet connections are becoming more affordable, promoting wider usage.^16^ Smartphones were the most popular device used by the preschool children in our study, different from previous results which found that children and youth mostly had access to laptops (46.3%), personal computers (21%) and tablets (15.3%). Smartphones appear to have become more popular over time, as they are more user friendly, easily portable, and more affordable than laptops.^19^

Almost all children in our study used digital devices under supervision by their parents or caregivers for the prevention of exposure to inappropriate content and guidance. However, socially desirable bias on self-reporting of supervision could not be excluded. Other studies also showed that parents did not allow their children to use digital devices independently owing to concerns about inappropriate contents or because their children requested for their _guidance._^6,10,20^

Although most preschool children used digital devices for approximately 30 minutes daily herein, half of them spent 1–2 hours on digital devices on weekends. This prolonged digital device use during weekends is a concern, as it exceeds the upper limit of the recommended duration for this age group (less than 1 hour per day) by twofold.^18^ The duration of digital device use on weekends in this study was higher than the average screen time of children in Hong Kong and America — 31 and 159 minutes, respectively.^3,10^ This needs immediate intervention, as prolonged screen time of 1–2 hours daily may cause language, behavioural, inattention and health problems, such as obesity.^21-23^ Since digital device use in this study was mostly supervised, the screen time could be moderated and replaced with involvement of parents in interactive family games and activities. Herein, the common reasons for allowing digital device use were educational and entertainment purposes. In the USA, parents allowed digital device use to keep their children out of trouble and safe, while in Greece, they allowed such as a reward for good behaviour or for learning.^6,24^ Some benefits of digital device use among preschool children include the development of fine motor skills with the use of touch-screens and symbols along with improvement of their skills and creative imagination.^20^

The strength of this study was that the description of digital device usage among young children was evaluated in detail. Meanwhile, the limitations were that the data were collected from a single population; hence, the findings may not be generalisable to the general population. Recall and socially desirable biases may have caused underreporting owing to perceived undesirable behaviour. The age for preschool children in Malaysia is 4-6 years, different from that in other countries. Further, the classification of preschool children was different; comparing the results among preschool children was thereby challenging. Some studies also included televisions as digital devices when measuring screen time, making it challenging to directly compare our results with previous data.

## Conclusion

We found a high prevalence (95.9%) of digital device use among preschool children, with an average age at first exposure of 3.9 years. The most popular digital device was smartphones, which were mainly used for entertainment and educational purposes. A high percentage (95.7%) of digital device use was under parental supervision. Although the digital device usage duration in most children was < 1 hour daily, approximately 20% and 50% of them spent more than the recommended screen time during weekdays and weekends, respectively. These findings could promote awareness on the high prevalence of digital device use among preschool children and on the exceeded recommended screen time during weekends. Accordingly, these could help design public health awareness programmes and future policies.
